# Influence of the Compaction Method in the Volumetric Design of Cold Recycled Mixtures with Emulsion

**DOI:** 10.3390/ma14051309

**Published:** 2021-03-09

**Authors:** Guillermo Flores, Juan Gallego, Lucía Miranda, José Ramón Marcobal

**Affiliations:** 1 Departamento de Ingeniería del Transporte, Territorio y Urbanismo, Universidad Politécnica de Madrid, 28040 Madrid, Spain; guillermo.flores1091@gmail.com (G.F.); jrmarcobal@sacyr.com (J.R.M.); 2 Repsol, 28045 Madrid, Spain; marialucia.miranda@repsol.com; 3 Sacyr, 28027 Madrid, Spain

**Keywords:** cold asphalt mixtures, recycled mixtures, Proctor, gyratory compactor, maximum bulk density

## Abstract

Compaction of cold asphalt mixtures is a subject that has not been thoroughly studied, and, for this reason, requires new efforts from researchers to have a better understanding. Unlike hot mixtures, cold mixtures and mainly recycled mixtures require specific considerations for compaction. There is a lack of consensus about the methodology to select the optimum premix water and emulsion contents. In the absence of specific regulations, the use of soil tests or hot mixtures procedures is common. For these reasons, this investigation’s main goal was to evaluate two compaction methods used to design cold recycled mixtures with emulsion: the modified Proctor procedure and the gyratory compaction. It was concluded that both methods could be useful to study compactability since consistent results were obtained by applying the maximum bulk density criterion. However, the higher bulk densities achieved, the smaller specimens used, and the suitability of the gyratory specimens to be later tested for mechanical properties make them preferable to the modified Proctor samples. A new approach has been proposed using iso-density lines on dual-axis premix water content-emulsion content graphs that facilitates the study of the influence on compactability of these two factors combined. These contributions can alleviate the laboratory works during the design of cold recycled asphalt mixtures and contribute to a more in-depth knowledge of the combined effect of premix water and emulsion contents on the compactability of cold recycled mixtures with emulsion.

## 1. Introduction

Asphalt mixture after production is a loose material, made of stone aggregate coated with a binder in a mortar matrix. Compaction is one of the most critical steps in the process of road construction, because of its significant influence in the short- and long-term performance of the pavement.

During compaction, aggregates are brought together, creating a skeleton that provides resistance to deformations and at the same time limits permeability by reducing air void content. The compaction process can be affected by many factors, such as asphalt binder and aggregates properties, mix type and particle size distribution, compaction temperature, lift thickness, underlying course properties and environmental conditions. Asphalt binder properties change with temperature, which means that there is a specific range where viscosity permits adequate compaction by providing lubrication between particles during the compaction process. Low temperature prevents aggregate particles from moving, and proper densification of the mixture is difficult to achieve [1].

Proper compaction means fewer particle re-orientations under the traffic loads. Fewer particle re-orientations also means less plastic deformations and rutting in the pavement. Furthermore, proper compaction ensures more inter-particle contact points, which results in smaller forces in the inter-particle contact points [2].

The asphalt pavement achieves the density established in the laboratory through sufficient compaction efforts in the field. Therefore, understanding the compaction characteristics of asphalt mixtures is of great importance [3].

In road engineering techniques, the maximum density of materials is a parameter introduced to obtain the material’s best mechanical behaviour under loading conditions. Compaction is intended to reduce air void content, optimise the granular skeleton and increase material density [4].

Even with the finest design or the highest quality materials, if the asphalt mixture was compacted improperly, under-compacted, or over-compacted, it will not offer the best pavement performance [5]. Improperly compacted asphalt pavement cannot transfer loads from the surface to the ground.

Some pavement distresses, such as rutting, water seepage and cracking, quickly appear in under-compacted asphalt pavement. Therefore, reasonably controlling compaction degree is the key to guarantee the healthy life of asphalt pavement [5].

The distribution of air voids also affects the presence and movement of water in asphalt mixtures. Water weakens the adhesive bond between the aggregates and the binders. The cohesive bond within the mastic itself leads to the asphalt mixture’s disintegration and, ultimately, to the pavement structure’s failure [2].

An asphalt mixture that exhibits proper compaction can be easily compressed to the volumetric design requirements while displaying stability, resistance to deformation and high-performance during road service life [6].

Additionally, the compaction process requires enough rollers, which are a significant source of carbon emissions in this area. Carbon emissions of these compaction methods are different and need proper assessment to meet the growing concerns and regulations to minimise the carbon footprint [7]. Thus, the optimisation of the field rollers’ movements has been addressed by the monitoring with Global Positioning Systems (GPS), which has contributed to the homogeneity of the density and the pavement’s mechanical properties [8]. Both in the laboratory and the field, controls are necessary to verify material void contents. Within a laboratory setting, the focus lies in reproducing in situ conditions as closely as possible using appropriate equipment [4].

There are several types of laboratory methods, including impact compaction, kneading compaction, gyratory compaction and rolling wheel compaction. However, different methods in the laboratory may manufacture identical samples regarding the density while exhibiting different mechanical properties [9].

Marshall Stability and volumetric properties of hot asphalt mixtures are significantly affected by the compaction temperature, and mixtures with the same nominal maximum aggregate size but different gradation types require different compaction levels to achieve the designed density [6].

Over the last 50 years, Marshall Asphalt Mix Designs, based on impact compaction, dominated the world paving industry. On the other hand, the Superpave mix design method brought new challenges and opportunities to the compaction process. Superpave has permitted a better understanding of the compaction process by introducing the Superpave gyratory compactor (SGC), which allows monitoring the specimen height after each gyration and provides a better simulation of compaction than previous compactors. Although there is a method for monitoring the specimen height after each blow with the Marshall hammer, the breakdown of aggregates remains as one of the main drawbacks of this compaction procedure [1].

Marshall and Superpave mixture designs are based mainly on volumetric parameters. Several studies have already questioned the traditional Marshall compaction method for not resembling the one practised in the field [10]. Nowadays, several countries’ primary compaction method is changing to Superpave gyratory compactor (SGC) [3].

The air void content is one of the most used parameters in the mix design. The air void content obtained for cold recycled mixtures has emulsion ranges between 10% and 20% [11].

The bituminous mixture’s behaviour during compaction and its influence during its laying, and its ultimate mechanical stability, can be simulated at the laboratory scale, through the continuous register of data about the percentage of compaction in relation to the number of cycles of the gyratory compactor [12]. The European standard EN 12697-10 [13] proposes three laboratory methods for the characterisation of the compactability of hot asphalt mixtures, including impact, gyratory and vibratory compactors [2].

Nevertheless, in the case of cold mixtures, and especially in cold recycled mixtures, there is not enough information on the compaction method’s influence on the final properties of the material. Some guidance about ultimate properties as permanent deformation or fracture toughness can be found in Annex B: Performance characteristic assessment (Informative) of EN 13108-31:2019 [14] Bituminous mixtures, Material specifications, Part 31: Asphalt Concrete with Bituminous Emulsion. Nevertheless, in situ recycling is not covered by this standard.

As for the mixture’s design, the choice is primarily based on bulk density, which has to be necessarily associated with a compaction method. The optimum binder content for this type of mixture is traditionally selected utilising the modified Proctor test, which is a test initially conceived for granular layers and soils.

Some references have been found in the literature for the laboratory compaction of cold recycled mixtures with the gyratory compactor. In the first instance, most of the researchers used the gyratory compactor with a constant compaction pressure of 600 kPa, an external angle of 1.25° and a gyration rate of 30 rpm [15].

In the research of Sangiorgi et al. [16], nine specimens were prepared for each of the mixtures with gyratory compaction at 180 gyrations. Each specimen had a dry mass of 4500 g and a diameter of 150 mm. The workability and volumetric properties of the mixtures were assessed by means of the compaction curves obtained during the gyratory compaction. The volumetric characterisation was then supported by the analysis of the air voids content of each specimen after compaction.

In other research [17], cylinder specimens of cold recycled mixtures with emulsion were prepared by SGC with 170 mm in height and 150 mm in diameter.

In the work of Graziani et al. [18], specimens were compacted utilising a shear gyratory compactor (SGC) adopting a 150 mm diameter mould and compaction energy of 180 gyrations. Since increasing total water content helps in reducing the final volume of the specimens, it should be as high as possible. Nevertheless, Kuna and Guttumukkala [19] decided to use 100 mm diameter gyratory moulds for specimen preparation.

On the other hand, authors such as Amouzadeh and Modarres [20] preferred the modified Marshall mix design method to design the cold recycled asphalt mixture. The specimens were compacted using the Marshall hammer by applying 50 blows to each side.

Dołżycki and Jaskuła [21] have determined the optimum moisture content for the tested mixes using the Marshall method, taking into account the water included in the bituminous emulsion as well as an additional wetting effect by the bitumen included in the emulsion. Six different combinations of cement and bituminous emulsion content were used. Specimens for testing were compacted in a Marshall compactor with 75 blows per side.

Flores et al. have studied the energy of compaction and the evaluation of the mixture’s mechanical properties. It has been possible to establish the energy to be implemented by the gyratory compactor to approximately achieve the field compaction level [22].

Finally, in the research of Valentin et al. [23], the cylindrical specimens of 150 mm diameter and 60 mm height were prepared by putting the cold recycled mix in cylindrical moulds and compacted by applying pressure of 5.0 MPa.

In the case of Spain, the specification for road rehabilitation PG4 [24] initially established the cold recycled mix design through the modified Proctor test. In 2017, the standard changed to require the gyratory compactor. Nevertheless, there are no study cases in the bibliography to allow engineers and practitioners to have proper knowledge of the direction and magnitude of this change in the official regulations.

For this reason, the present study evaluates the influence of the compaction method in the dosing of cold recycled mixtures with emulsion, considering the compaction with the modified Proctor test and the gyratory compaction, according to the old and new version of the Spanish regulations. The objective is to elaborate a comparative study of the Proctor/gyratory compactor on a series of working formulas that will be compacted and studied by both methods. The comparison will consider density and air voids. The investigation’s ultimate target is to make clear the suitability of both compaction methods for the design of cold recycled mixture with bituminous emulsions.

## 2. Materials

A cold mix with emulsion was manufactured with reclaimed asphalt pavement (RAP). The RAP granulometry fits the RE2 band recommended by Spanish regulations PG4 [24], as shown in Table 1. No virgin aggregate was added.

The bitumen was extracted from the RAP to know its properties. As presented in Table 2, the bitumen was in aged condition after years in service.

A commercial cationic bituminous emulsion recommended for cold recycling applications, with a residual bitumen content of 60%, slow breakage B5, produced with rejuvenating agents (C60 B5 REC REJUV), was used to produce the laboratory specimens.

## 3. Methodology

This investigation evaluates the influence of compaction methods on the volumetric properties of cold recycled asphalt mixtures with emulsion. Proctor and gyratory compaction methods were performed to compare the results.

For this study, 16 different formulas were tested, including four different percentages of emulsion (1%, 2%, 3%, 4%) and four different premix water percentages (1%, 2%, 3%, 4%). These emulsion ranges were selected to meet article 20.3 of PG4 Spanish specifications for cold recycling with emulsion [24]. The premix water range was proposed according to the observations from the first trials in the investigation. Values of premix water above 4% showed excessive water drainage during compaction.

### 3.1. Compaction Procedures

For the modified proctor test (UNE 103501-94 [28]), approximately 5 kg of RAP were needed for a specimen compacted in the standardised mould (152 mm diameter and 127 mm height). The compaction was carried out by impact, with a calibrated rammer of 4.535 kg and a standard falling height of 457 mm, in a universal Proctor compactor manufactured by Mecánica Científica S.A. After mixing, the specimens were compacted in five layers with 60 impact blows/layer on the layers’ upper surface. Once the compaction was completed, the extension of the mould was removed to eliminate the surplus material. The objective was to weigh the material’s mass inside the mould and then calculate the wet density (ρw), using the mould’s known volume. For each dosage, two specimens were manufactured, and the result was obtained as the average of them.

In gyratory compaction, a much lower amount of material is needed to produce a specimen. Since the specimens produced are 100 mm in diameter and between 60 and 70 mm in height, 1.1 kg of RAP was required to fill the mould. After mixing, the material was compacted to 100 gyrations, the compaction energy recommended by Spanish regulations for cold recycled materials with emulsion. The compaction was carried out with an internal rotation angle of 0.82°, a speed of 30 rpm and a maximum pressure of 600 kPa, in a gyratory compactor manufactured by Cooper Research Technology Limited. Two specimens were manufactured for each dosage, and the result was calculated as the average.

It should be noted at the outset that the gyratory procedure offers some advantages. The amount of material needed to perform the modified Proctor procedure is five times higher than the procedure’s material with the gyratory compactor. Moreover, the specimens obtained by the gyratory compactor, 100 mm in diameter and 60–70 mm in height, can be used later to test the mixture’s mechanical properties: indirect tensile stress, stiffness modulus, etc. On the other hand, the modified Proctor specimens cannot be used after the bulk density has been calculated, as the process implies the deterioration of the specimen.

### 3.2. Maximum Density

**Figure 1:**
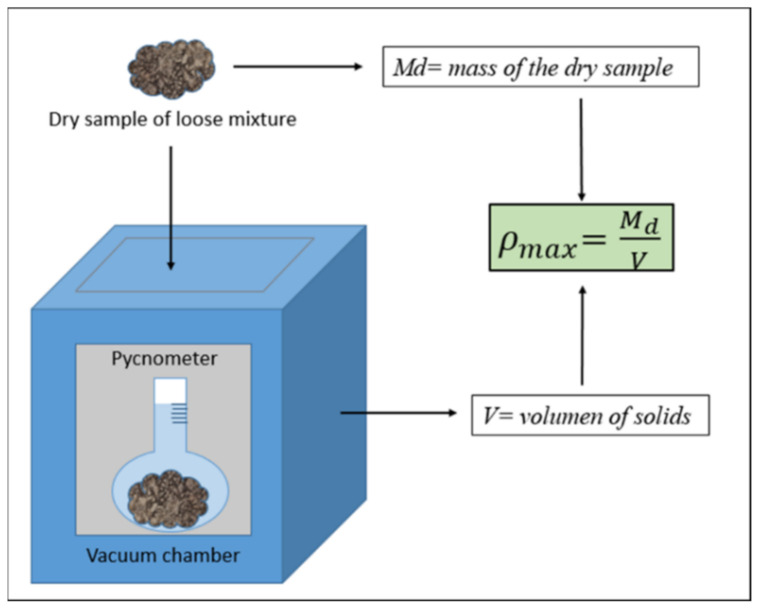
Scheme of the calculation of maximum density, ρ*_max_*. Valid for Proctor and gyratory procedures.

The maximum density is a theoretical concept that represents the material without air voids. That would mean that the specimen contains only dry material accommodated so that the air voids content is zero. The maximum density is calculated with the pycnometer (Figure 1), which allows us to know the volume without air in the mixture. As the dry mass, Md, is known, the maximum density can be calculated (EN-12697- [29]).

### 3.3. Bulk Density and Air Voids Content

**Figure 2:**
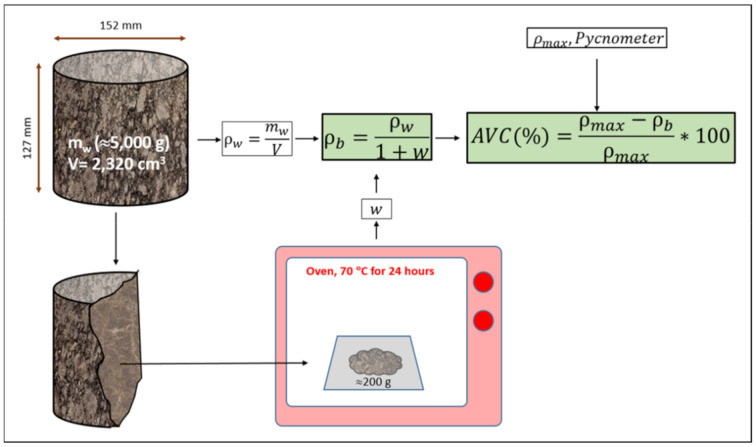
Scheme of the calculation of the bulk density and the air voids content for Proctor specimens.

Once the Proctor compaction is finished, the mould was weighed with the material (Figure 2). After that, a core 100–200 g was extracted from the centre of the specimen. This operation is carried out in order to weigh it and then put it in the oven to eliminate moisture. The drying process was performed at 70 °C for 24 hours. Then, the core sample was reweighed to obtain the moisture content. From the moisture content and the wet density (weight of the sample/volume of the mould), it is possible to calculate the compaction’s bulk density using Equation (1):

(1) $$\rho_{b}=\frac{\rho_{w}}{\left(1+w\right)}$$

where ρ*_b_* is the bulk density, ρ_w_ is the wet density and w is the moisture measured in the sample.

The air void content was calculated through the bulk density and the maximum density, following Equation (2).

(2) $$Air\;void\;content\;\left(\%\right)\;=\frac{\rho_{max}-\rho_{b}}{\rho_{max}}\times 100$$

where ρ*_max_* is the maximum density and ρ*b* is the bulk density.

**Figure 3:**
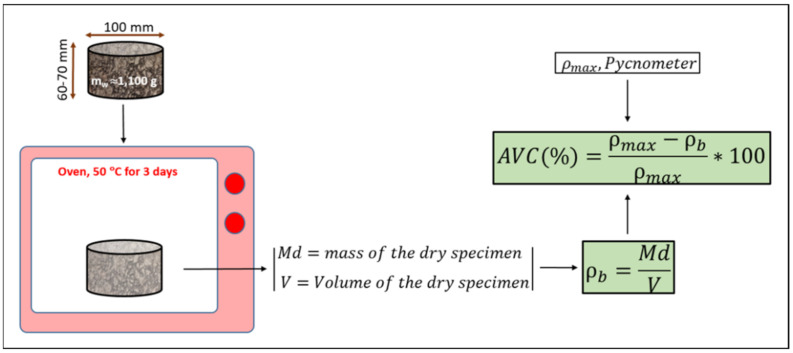
Scheme of the calculation of the bulk density and the air voids content for gyratory specimens.

In the gyratory specimens, the volume was calculated by the diameter and height of the sample after compaction (Figure 3). As the bulk density corresponds to the dry material, it was necessary to determine the moisture, w, by putting the entire compacted samples in the oven to evaporate all the humidity, which was achieved after three days at 50 °C.

## 4. Results

The following sections present the results obtained in the laboratory.

### 4.1. Maximum Density of the Mixtures

The results obtained are shown in Table 3. The nomenclature used to identify every formula consists of the letter W followed by the premix water content and the letter E followed by the emulsion content. The percentage of water and emulsion are expressed by weight of RAP. In this way, the identification of each formula is easy.

### 4.2. Bulk Density and Air Voids of the Mixtures

The results of the bulk density and the air voids content for each mixture compacted by the Proctor and gyratory method were calculated through Equations (1)–(2). The results can be seen in Table 4 and Table 5.

## 5. Analysis of Results

### 5.1. Analysis of Conventional Graphs for Bulk Density and Air Voids

**Figure 4:**
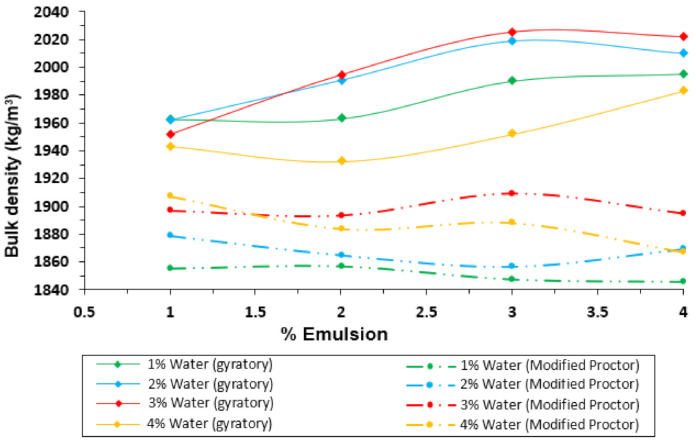
Comparison of bulk density for different premix water contents in samples compacted with the gyratory compactor and the modified Proctor procedure.

**Figure 5:**
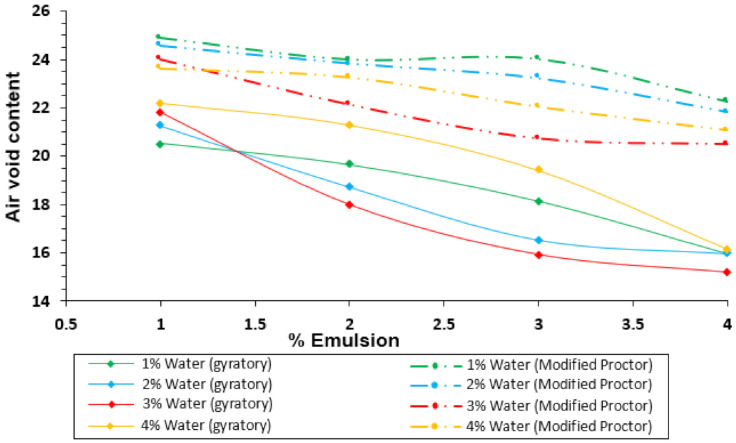
Comparison of air voids content for different premix water contents in samples compacted with the gyratory compactor and the modified Proctor procedure.

Figure 4 shows the evolution of the bulk density (y-axis) obtained with the gyratory compactor and the modified Proctor procedure over the emulsion content (x-axis), for every premix water content added to the mixture. Similarly, Figure 5 shows the comparison of these two methods for the air void content.

Several considerations can be made from the results displayed in Figure 4 and Figure 5. In the first instance, the bulk densities reached with the gyratory compactor are higher than those obtained by the Proctor procedure. Nevertheless, both compaction methods show similar tendencies (air voids content decreasing when increasing % of emulsion). It is possible to notice that the compaction with both methods starts with similar air voids values for low emulsion contents, but the gyratory graphs’ slope is the highest. These different slopes could indicate that the gyratory method is more sensitive to emulsion content than the Proctor procedure.

**Figure 6:**
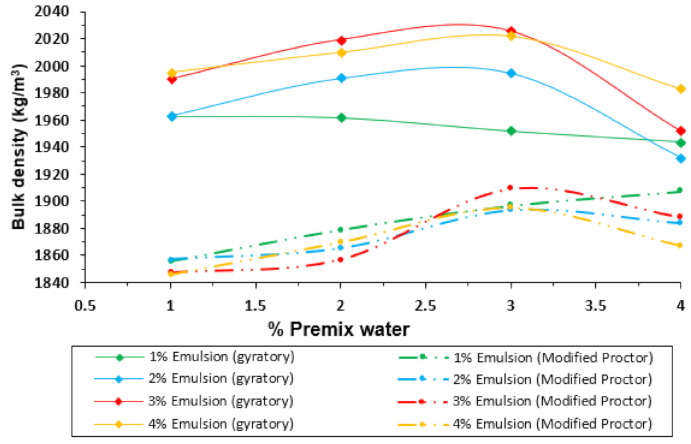
Comparison of bulk density for different emulsion content in samples compacted with the gyratory compactor and modified Proctor procedure.

**Figure 7:**
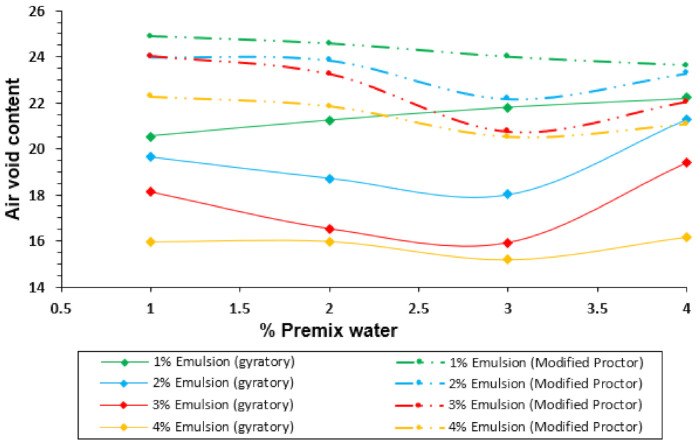
Air void content for different emulsion content in samples compacted with the gyratory compactor.

Figure 6 and Figure 7 display another perspective of the results with the gyratory compactor and the modified Proctor procedure. In this case, the bulk density and the air void content are represented on the y-axis, and the premix water on the x-axis, as usual when presenting the results for the Proctor compaction of soils with no emulsion.

In the compaction with the gyratory method, it is possible to notice that the premix water content improves the compactability in most cases. For this reason, a decrease of the air void content can be noticed from 1% to 3% of premix water. Nevertheless, 4% of premix water results in the worst performance, reaching even higher air-void content values than for 1% of premix water. This phenomenon can be explained as the water reaches a maximum (optimum) point to which the compactability grows because the water facilitates the aggregates’ accommodation. However, when that point is exceeded, the premix water stops working as a lubricant and it begins to contribute to the resistance to compaction.

As for the Proctor compaction, it must be noted that, as in the case of the gyratory compactor, there is optimum water content. From 1% to 3%, there is an increase in compactability, but for 4% of premix water content, the compaction achieved decreases. In this case, the decrease is not as pronounced as it was with the gyratory compaction, because 4% of water does not reach air voids content above the values for 1% premix water. This difference in the gyratory samples can be explained because there is no confinement in the sample’s upper side, which drastically prevents the free movement of excess water in the material. The Proctor hammer is applied every time in a limited area at the top of the layer. Water opposes less resistance to compactability than in the case of the gyratory compactor, which creates pressure on the entire top side of the specimen.

In both methods, the Proctor and the gyratory compactor, the minimum air voids content and maximum bulk density for the mixture studied is located at 3% of premix water. It is important to note that there is independent optimum premix water content in all the figures presented above, regardless of the emulsion content, except for the very scarce content of 1% emulsion (Figure 6). The optimum for the emulsion content can be found in both methods, paying attention to the 3% premix water content (Figure 4) and selecting the content emulsion corresponding to the maximum bulk density. In both cases, the solution is 3% of emulsion content.

### 5.2. Proposal of a New Approach for the Volumetric Design of Cold Recycled Mixtures with Emulsion

According to the results of this investigation, both methods, the gyratory compactor and the modified Proctor procedure, can be useful for studying compactability and designing the mixture, since consistent results are obtained.

Nevertheless, for any of the two methods under comparison, the fact that there are two variables—premix water and emulsion contents—makes the process difficult compared to the more straightforward selection of the optimum moisture in soil-water systems.

**Figure 8:**
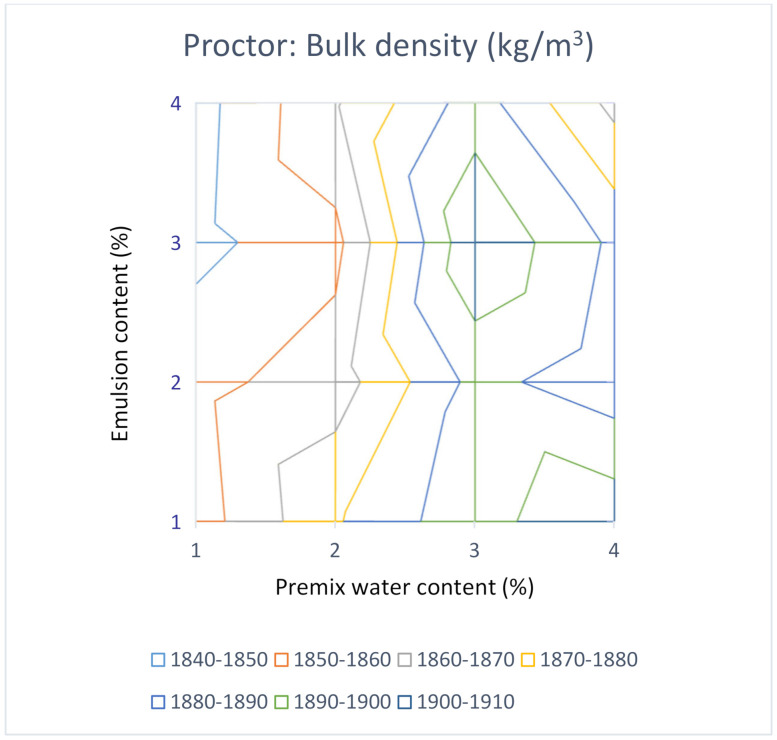
Bulk density for mixtures compacted with the modified Proctor procedure.

**Figure 9:**
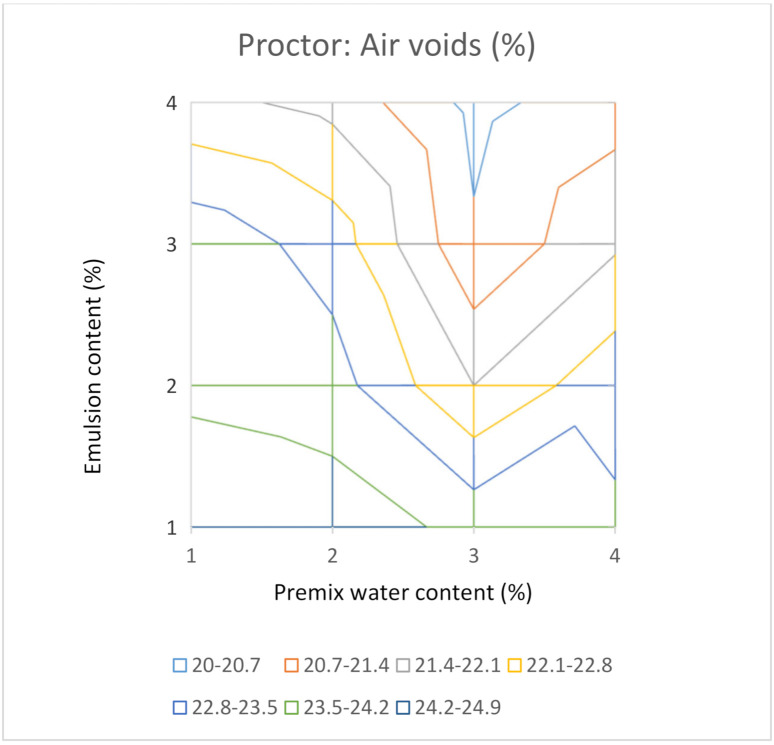
Air voids content for mixtures compacted with the modified Proctor procedure.

Indeed, it would be desirable to test four contents of premix water and four contents of emulsion, making a total of sixteen combinations. As two samples are compacted for each combination, to calculate an average value, a total of thirty-two samples would be necessary for a complete study. This group of values allows us to elaborate a surface graph with iso-density lines or iso-air void content to make all the laboratory results comprehensible at a glance. Figure 8 and Figure 9 show these types of graphs for the Proctor compactor’s results in this investigation.

It can be seen in Figure 8 that the solution is near 3% of premix water and 3% of the emulsion by the criterion of maximum bulk density. It is probably the optimal combination of water and emulsion for the RAP under study from a volumetric perspective. It is worthy to note that per the representation of air voids content in Figure 9, the minimum for this parameter corresponds to the combination of 3% water and 4% emulsion. The optimal combination of the maximum bulk density is not coincident with the optimal combination when applying the criterion of minimum air voids content. This apparent contradiction can be explained by Equation (2), which is the mathematical expression of the air voids content. For simple soil-water systems (with no emulsion), the highest bulk density, ρ*b*, corresponds to the lowest air voids content, as the maximum density, ρ*max*, does not depend on the emulsion content. Nevertheless, in RAP water-emulsion systems, the maximum density, ρ*max*, decreases as the emulsion content increases. For this reason, the lowest value for air voids content is not necessarily associated with the higher value of the bulk density, in contrast to what occurs in the case of soil-water systems.

**Figure 10:**
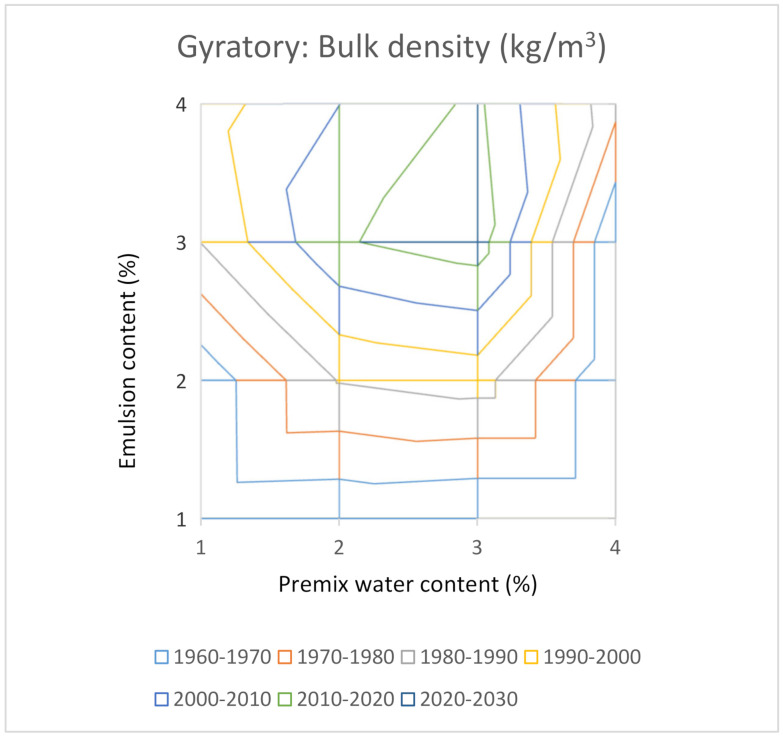
Bulk density for mixtures compacted with the gyratory compactor.

**Figure 11:**
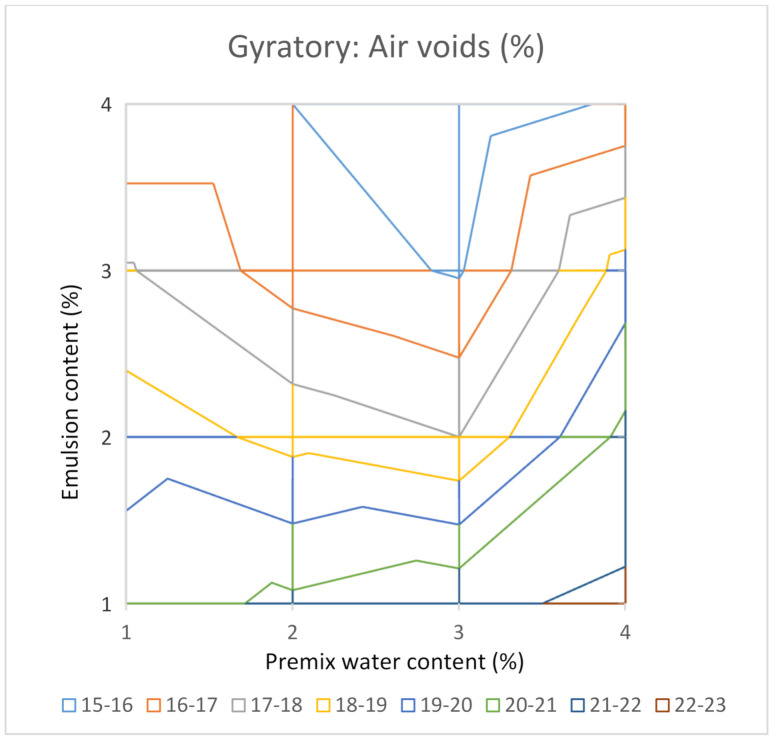
Air voids content for mixtures compacted with the gyratory compactor.

The results of the study by the gyratory compactor are plotted in Figure 10 and Figure 11. The maximum for the bulk density (Figure 7) corresponds to the combination that includes 3% of premix water and 3% of the emulsion. As in the Proctor compaction case, this optimum is not coincident with the optimum by the criterion of minimum air voids (Figure 11). The explanation is the same as that in the precedent case of the Proctor compaction.

With this analysis of results, based on bulk density iso-lines, it is evident that the bulk density offers a maximum value inside the graph. This makes it possible to consider this graph a useful tool to select the optimum formula from the volumetric point of view. Nevertheless, the air voids content decreases as the emulsion content increases, and the minimum value would be located outside the graph, and probably would correspond to excessive content of the emulsion.

Regarding the method of compaction, there are several reasons to prefer the gyratory compactor. First of all, the bulk densities obtained seem to be nearer the field experience. Additionally, it is worth mentioning that for a complete study, 32 samples are required. In terms of the modified Proctor procedure, the total amount of materials would include approximately 160 kg of RAP and 5 kg of emulsion. In the case of the study with the gyratory compactor, 35 kg of RAP and 1 kg of emulsion would be necessary. Moreover, as Flores et al. [11] proposed, the specimens obtained by the gyratory compactor, 100 mm in diameter and 60–70 mm in height, can be used later to test the mechanical properties of the mixture: indirect tensile stress, stiffness modulus, etc. Nevertheless, the modified Proctor specimens cannot be used after the bulk density has been calculated, as the process implies the deterioration of the specimen.

## 6. Conclusions

The present work aimed to define the best methodology for the volumetric design of a cold recycled mixture with emulsion. A comparison between the modified Proctor procedure and the gyratory compactor has been carried out based on the bulk density and the air void content of the mixtures under study.

It is useful to graph the results in a surface graph with iso-bulk density lines to understand at a glance the evolution of the bulk density with the contents of premix water and emulsion. In both cases, gyratory and modified Proctor, a maximum for the bulk density was obtained inside the area analysed. Nevertheless, when the results for the air voids content were plotted in the air voids iso-lines graph, it could be observed that the combination of premix water and emulsion for the minimum air voids content is not coincident with that for the maximum bulk density. This apparent contradiction can be explained by the fact that the air voids content depends on both the maximum density and the bulk density. The highest bulk density corresponds to the lowest air voids content for simple soil-water systems, as the maximum density does not depend on the emulsion content. Nevertheless, in the case of RAP water-emulsion systems, the maximum density decreased as the emulsion content increased.

For this reason, the lowest value for air voids content is not necessarily associated with the higher value of the bulk density, in contrast to what occurs in the case of soil-water systems. The lowest air void content is not a valid criterion to select the optimal formula for the mixture. The application of this wrong criterion could result in excessive contents of the emulsion detected in a second phase of the design that includes tests for the mixtures’ mechanical performance.

In this study, sixteen combinations (1%, 2%, 3% and 4% of premix water with 1%, 2%, 3% and 4% of emulsion) were manufactured. The bulk density iso-lines analysis allowed us to find the maximum value, which has been adopted as the criterion to select the optimal mix formula.

The analysis of iso-bulk density lines indicated that the gyratory compactor achieves bulk densities higher than those obtained by the modified Proctor procedure. Nevertheless, both the gyratory compactor and the modified Proctor procedures resulted in the same optimal formulation for the maximum bulk density, being 3% of premix water and 3% of the emulsion.

Nevertheless, regarding the compaction procedure to perform this methodology of study, the gyratory compaction of specimens with 10 mm diameter and 60–70 mm height seems to be preferable to the modified Proctor procedure, based on the following reasons: (1) the bulk densities obtained were higher and closer to the field experience, (2) according to the results of this research, the gyratory curves for the bulk density seem to be more sensitive to the changes in the formulation than the modified Proctor curves, (3) the amount of material needed to perform the modified Proctor procedure is five times higher than the material used for the procedure with the gyratory compactor and (4) the samples obtained by the gyratory compactor, 100 mm in diameter and 60–70 mm in height, can be used later to test the mechanical properties of the mixture: indirect tensile stress, stiffness modulus, etc. On the other hand, the modified Proctor samples cannot be used after the bulk density has been calculated, as the process implies the sample’s deterioration.

Based on the above, the authors recommend the use of the gyratory compactor to determine the maximum bulk density obtained for a group of combinations of premix water and emulsion contents as the best criterion to define the optimal mix formula. The use of surface graphs with iso-bulk density lines to better understand the mixture’s compaction performance is highly recommended.

These findings can help facilitate the laboratory works during the design of cold recycled asphalt mixtures. Moreover, this investigation contributes to a more in-depth knowledge of the combined effect of premix water and emulsion contents on the compactability of cold recycled mixtures with emulsion.

Finally, it should be noted that this research did not have field data to establish a relationship between the density obtained in full-scale projects and that obtained in laboratory specimens with the Proctor or gyratory compactor. Clarifying this aspect is of utmost importance for the advancement of this technique and should be the next step in this research line.

## Figures and Tables

**Table 1:** Granulometric band RE2 (PG4). % Passing.

Sieve (mm)	20	12.5	8	4	2	0.5	0.25	0.063
RAP curve	100.0	75.0	63.0	44.0	27.0	10.2	6.6	2.7
Minimum	80.0	62.0	49.0	31.0	19.0	2.0	0.0	0.0
Maximum	100.0	89.0	77.0	58.0	42.0	20.0	10.0	3.0

**Table 2:** Properties of the recovery binder.

Properties	Normative	Values
Bitumen (% by weight of RAP)	EN 12697-1 [25]	4.89
Softening Point (℃)	EN 1427 [26]	80.3
Penetration (10^−1^mm)	EN 1426 [27]	11

**Table 3:** Maximum density of the mixtures studied.

Mixture	Maximum Density (kg/m^3^)
W1E1	2470
W1E2	2443
W1E3	2431
W1E4	2374
W2E1	2491
W2E2	2449
W2E3	2419
W2E4	2392
W3E1	2496
W3E2	2432
W3E3	2409
W3E4	2384
W4E1	2498
W4E2	2455
W4E3	2422
W4E4	2365

**Table 4:** Bulk density and air void content for mixtures by the modified Proctor test.

Mixture	Bulk Density (kg/m^3^)	Air Void Content (%)
W1E1	1855	24.9
W1E2	1857	24.0
W1E3	1847	24.0
W1E4	1846	22.3
W2E1	1879	24.6
W2E2	1865	23.8
W2E3	1857	23.2
W2E4	1869	21.9
W3E1	1897	24.0
W3E2	1893	22.1
W3E3	1909	20.8
W3E4	1895	20.5
W4E1	1907	23.6
W4E2	1884	23.3
W4E3	1888	22.0
W4E4	1867	21.1

**Table 5:** Bulk density and air void content for mixtures by the gyratory compaction.

Mixture	Bulk Density (kg/m^3^)	Air Void Content (%)
W1E1	1962.7	20.5
W1E2	1963.0	19.6
W1E3	1990.2	18.1
W1E4	1995.3	16.0
W2E1	1961.8	21.2
W2E2	1990.6	18.7
W2E3	2019.1	16.5
W2E4	2009.9	16.0
W3E1	1951.5	21.8
W3E2	1994.4	18.0
W3E3	2025.3	15.9
W3E4	2021.9	15.2
W4E1	1943.4	22.2
W4E2	1932.2	21.3
W4E3	1951.9	19.4
W4E4	1983.0	16.2

## Data Availability

The data presented in this study are available on request from the corresponding author.
